# The Effect of Partial Corticotomy on the Rate of Maxillary Canine Retraction: Clinical and Radiographic Study

**DOI:** 10.3390/molecules25204837

**Published:** 2020-10-20

**Authors:** Hosam Ali Baeshen

**Affiliations:** Department of Orthodontics, Faculty of Dentistry, King Abdulaziz University, P.O. Box 80209, Jeddah 21589, Saudi Arabia; habaeshen@kau.edu.sa; Tel.: +966-55-460-0023

**Keywords:** anchorage, canine retraction, corticotomy, orthodontic tooth movement

## Abstract

The study aimed to evaluate, clinically and radiographically, the effect of partial corticotomy of the buccal plate distal to the canine on the rate of maxillary canine retraction. A clinical trial with the split-mouth design was conducted among twenty orthodontic patients, recommended for first premolar extraction with an age range from 13 to 21 years, selected from patients seeking orthodontic treatment in private dental clinics in Jeddah, Kingdom of Saudi Arabia. After extraction of the maxillary right and left first premolar, partial corticotomy was performed distal to the canine on the right side. The canine retraction was carried out with a power chain on both sides extended between the canine and the maxillary first molar. The data collected from the current study were tabulated and statistically analyzed using an independent sample *t*-test with *p* < 0.05 considered statistically significant. The rate of canine retraction was significantly higher on the corticotomy side than the control side (*p* < 0.05). Under the limitations of the present study, it can be concluded that the technique of partial corticotomy of the buccal plate distal to the canine is a straightforward surgical procedure enhancing the rate of canine retraction significantly.

## 1. Introduction

Orthodontic tooth movement is achieved by the application of a force to induce bone resorption on the pressure side and bone apposition on the tension side [1,2,3]. Classically, the rate of orthodontic tooth movement depends on the magnitude and duration of the force, the number and shape of the roots, the quality of the bony trabeculae [4,5,6,7], the patient’s response, and the patient’s compliance. The rate of biologic tooth movement with maximum mechanical force is approximately 1–1.5 mm in 4–5 weeks [8]. Therefore, in the maximum anchorage premolar extraction cases, canine distalization usually took 6–9 months, contributing to the overall treatment time of 1.5–2 years.

The longer orthodontic treatment duration implies a greater risk for the patient. The risk of orthodontic treatment includes enamel demineralization, caries, periodontal disease, and root resorption [9]. An increase in the duration of the applied force has been associated with increased root resorption [10]. It is generally accepted that the best way to minimize root resorption is to complete the tooth movement in a short time. Root resorption begins 2–3 weeks after the orthodontic force is applied and continues for the duration of the force application [10,11,12]. Moreover, any technique that takes longer than three weeks to retract a canine will result in the loss of anchorage [13]. Moreover, the duration of the orthodontic treatment is one of the things that orthodontic patients complain about, especially adult patients [14].

Many attempts have been made to shorten the orthodontic treatment duration. These techniques can be summarized into three major groups [15,16], namely biologic approaches or the local administration of chemicals, physical or mechanical stimulation of the alveolar bone, using lasers, piezoelectric, direct electrical current, or magnets, and surgical techniques, including dental distraction and alveolar corticotomies. Among these, partial buccal corticotomy distal to the canine has shown great promise for the achievement of rapid canine retraction. Also, the accelerated osteogenic orthodontics technique (AOO) was reported to offer eight years of stability and rapid tooth movement.

**Figure 1:**
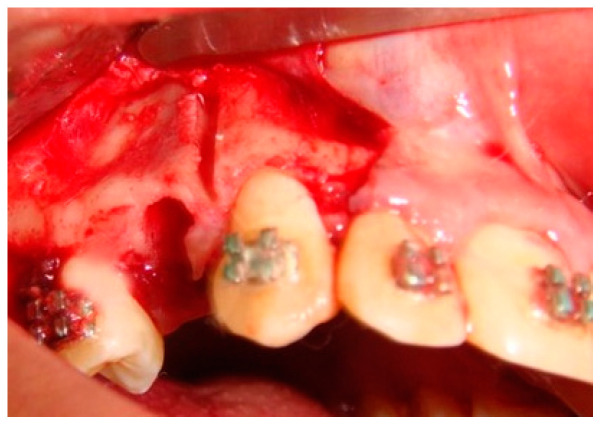
Surgical site partial corticotomy.

Based on the concept that teeth move faster when the resistance exerted by the surrounding cortical bone is reduced, Köle introduced a surgical procedure involving both osteotomy and corticotomy to accelerate orthodontic tooth movement [17]. Numerous studies have confirmed the usefulness of the corticotomy to accelerate orthodontic tooth movements. However, most of these studies have been conducted on animals [16,17,18,19,20]. A few studies on human subjects have also revealed the merit of this technique [21,22,23]. Therefore, an extensive review of the literature reveals that it is worth exploring the hypothesis that partial buccal corticotomy distal to the canine could enhance canine retraction and avoid the undesirable side effects of canine distraction techniques. Figure 1 offers a representation of clinical image for a better understanding of inside partial corticotomy.

Hence, this study aimed to evaluate, clinically and radiographically, the effect of partial corticotomy of the buccal plate distal to the canine on the rate of maxillary canine retraction. The null hypothesis tested in the study was that there is no difference in the rate of maxillary canine retraction between the experimental (partial buccal corticotomy) and control (no surgery) side.

## 2. Results

The current study was performed on twenty patients (two male and eighteen females) with an age range of 13–21 years, with a mean age of 16 ± 2.8 years. Two patients were excluded from the study as they failed to maintain several consecutive appointments. However, these two patients continued up to four months of treatment, so they were included in calculating the rate of canine retraction. The patients reported no complications during the healing of the partial buccal corticotomy side.

### 2.1. Rate of Canine Retraction

**Figure 2:**
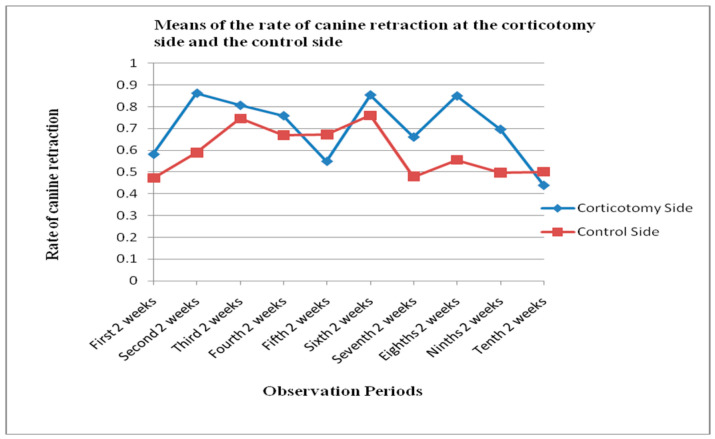
Rate of canine retraction at the partial corticotomy side and the control side during the observation periods of the study (every two weeks) as measured clinically (mm/week).

The rate of canine retraction was measured clinically and was calculated immediately after complete retraction of the canine at either side, experimental or control. The rate of canine retraction at the experimental and control side as measured clinically was not different significantly than when measured on the study cast. The canine retracted in the partial buccal corticotomy side at a rate of 0.70 ± 0.14 mm/week, which was significantly higher than the rate of canine retraction at the control side, which was 0.59 ± 0.11 mm/week *p* = 0.01 (Table 1) The duration required for the canine at the corticotomy side to be completely retracted ranged from 14–20 weeks (Figure 2).

### 2.2. Molar Anchorage Loss

The mean anchorage loss at the experimental side was 0.91 ± 0.49 mm when measured from the mesiobuccal cusp of the upper first molar to the tangent at the distal surface of the second molar (first measurement) and 1.75 ± 0.61 mm when measured from the mesiobuccal cusp of the upper first molar to the tangent to the labial surface of the central incisors (second measurement) while it was 1.00 ± 0.54 mm and 2.0 ± 0.7 mm for the control side, respectively. There was no statistically significant difference in the molar anchorage loss between the experimental and the control side (Table 2).

### 2.3. Premolar Anchorage Loss

The average total anchorage loss of the upper second premolar is given in Table 3. The anchorage loss at the buccal partial corticotomy side was 0.75 ± 0.68 mm when measured from the intersection of the central groove to the line joining buccal and palatal cusp tips to the tangent of the upper surface of the third palatal rugae (first measurement) and was 1.50 ± 0.7 mm when measured from the intersection of the central groove to the line joining buccal and palatal cusp tips to the tangent of the labial surface of the upper central incisors (second measurement) while the anchorage loss was 1.28 ± 1.09 mm and 2.00 ± 0.95 mm at the control side respectively. The premolar anchorage loss in experimental and control sides was not statistically significantly different with both methods of measurement (Table 3).

### 2.4. Canine Rotation

The mean of upper canine rotation in distopalatal direction was 15.16 ± 6.67 degrees in the partial buccal corticotomy side and 18.16 ± 5.91 degrees in the control side. The difference between them was not statistically significantly different (Table 4).

### 2.5. Molar Rotation

The upper 1st molar rotation mean was 6.00 ± 1.41 degrees for the experimental side and 6.33 ± 1.75 degrees for the control side. The difference between them was not statistically significant (Table 5).

### 2.6. Bone Density

The bone densities were measured in Hounsfield units (HU). The difference in densities at the corticotomy side and the control side, both mesially and distally was not statistically significant (Table 6).

### 2.7. Root Resorption

Minimal to no root resorption was detected on the digital periapical radiographs in both experimental and control sides

### 2.8. Pocket Depth

There was no statistically significant difference observed in the pocket depth between the experimental and control side (Table 7).

## 3. Discussion

The present study was conducted to evaluate the effect of partial corticotomy of the buccal plate distal to the canine on the rate of maxillary canine retraction. It was observed that the rate of canine movement was significantly higher in the experimental side when compared to the control side. Hence, the null hypothesis was rejected.

Conventional orthodontics has always been the treatment of choice for class I bimaxillary protrusion and angle class II Div I. However, the need for accelerating the treatment progress to meet the patient’s expectations during the intermediate phases of the therapy and the need of anchorage control resulted in the combination of orthodontic treatment with a surgical procedure. Therefore, alveolar corticotomy with the conventional method was proposed as an alternative method to conventional orthodontic treatment, especially in difficult adult cases for rapid orthodontic tooth movement [13,14,24,25,26].

Concerns about the possible risks of the corticotomy procedure prompted the modification of this technique. The original technique was described and included a combined interradicular corticotomy and supra-apical osteotomy [14]. Although the results of the Kole [17] osteotomies were stable, pulp mortifications were not rare. Later, the supra-apical osteotomy was replaced by corticotomy, and labial and lingual corticotomy cuts were used to circumscribe the roots of the teeth. Generally, the conventional corticotomy techniques included both labial and lingual cuts and sometimes required two-stage surgery. Although the clinical healing was uneventful, some complications such as subcutaneous hematomas of the face and the neck could occur after intensive corticotomies [13,14,17,25,27,28].

In the current study, in which the buccal partial corticotomy technique was observed, the lingual vertical and subapical cuts were not performed, and the lingual flap was not elevated. The surgical procedure in the present study was different than the methods used in studies for the distraction of the periodontal ligament [26] since they made vertical grooves inside the extraction socket along the buccal and lingual sides and extended obliquely towards the socket base.

In contrast to Iseri’s [14] surgical work, only grooving of the buccal bone was performed in our study instead of complete removal with an exclusion distractor device.

The primary purpose of this conservative, one-stage surgery performed in the present study was to reduce the operation time and postoperative patient discomfort. Subsequently, no complications occurred. The healing in the present study was at least equivalent to the results obtained from similar studies [13,14,25,26]. In the current study, there was a minor amount of pain the first day after the surgery, followed by an increase in pain 2–3 days after the surgery, and a complete decline in pain after that in all subjects. However, the pain was not severe to interfere with the daily activities.

The average pain time was 2–4 days, in agreement with the results of Proffit [29] and Ngan et al. [30].

In evaluating the rates of canine movement in the present study, extrapolation of the velocities revealed that the rate of canine retraction at the experimental side by the corticotomy assisted canines retraction was significantly faster than that of the control side. The more rapid rate of canine retraction at the corticotomy side might be related to the reduced bone mass distal to the canine tooth. Accordingly, the time needed for overall cellular resorption in the pressure side might be reduced. The decreased bone density at the corticotomy side, as evident from the results of the current study, might also explain the increased rate of canine retraction at the corticotomy side. This might be explained in view of the results of Iseri et al. [14], who observed an increased rate of tooth movement in conjunction with increased marrow spaces which might stimulate the osteoclastic activity. This was similar to the findings reported by Liou et al. [25], Kisnisci et al. [27], and Sayin et al. [28].

However, the rate of canine retraction at the corticotomy side in the present study was faster than the conventional side, which was less when compared to the results of Iseri et al. [14], Liou et al. [13], Sayin et al. [28], and Kisnisci et al. [27]. This might be related to the massive amount of bone removed in their studies as compared to the current research, in which a small cut in the buccal cortical plate was made, leaving the original bony architecture intact.

Another factor that might contribute to the decreased rate of canine retraction as compared to the other studies was the use of the elastic chain which showed a sudden decrease of the force magnitude after the first 12 h, while the other studies [4,27,28,31] used a canine distractor which produced more steady, continuous, and massive force throughout the total distance of canine retraction.

The anchorage loss reported in this study was found to be minimal; fifty per cent of first molars in this study experienced less than 1.75 mm anchorage loss. The most considerable amount of anchorage loss was found to be 2.00 mm. This is in agreement with the findings of Liou and Huang [25] and Iseri et al. [14]. The former authors stated that 73% of the first molars did not move mesially whereas 27% moved less than 0.5%. They attributed this minimal amount of anchorage loss to the fact that the canines were rapidly retracted while the molars were still in the lag phase of tooth movement. The lag phase was the second phase of tooth movement wherein tooth movement stopped until the hyalinized areas were removed by undermining resorption and typically lasted for 2–3 weeks [25].

The minimal anchorage loss in the present study might be attributed to many factors for controlling anchorage units. These factors included: (a) the uses of transpalatal arch, (b) the inclusion of upper second premolar and upper first molar, and at the same time keeping the force magnitude on them very low, and (c) using the elastic chain might not be high enough to initiate molar tooth movement. Also, the role of corticotomy cannot be undermined in decreasing the resistance of canine for movement and substantially the force withstood by the anchorage units. Using mini-implants on the control side or corticotomy for canine retraction has shown no significant loss to the molar anchorage, as reported by Aboul-Ela et al. [21].

The retracted canine at both the control and experimental sides showed distopalatal rotation of 8 to 25 degrees with a mean of 15 degrees which might be related to the absence of lingual control on the canine during this retraction. This explanation was also described by Liou and Huang [25]. In this study, lingual elastics were not used due to that lingual traction applied to the maxillary canine during the initial stages of retraction might cause tipping of the canine around the lingual cortical plate of the alveolar process. This tipping might cause the root apex to come into proximity with the buccal cortical plate, rendering its uprighting and torqueing alignment extremely difficult [26]. Furthermore, root apex proximity to cortical plate predisposed the root apex to resorption [32]. Yet, the reported canine rotation degree in this study found to be higher than Rajcich and sadowsky [33].

The relatively minor amount of canine rotation reported in this study was not expected to present significant problems during the correction. The correction of the rotation after retraction was not time-consuming and did not place excessive demands on anchorage [34]. For the first molar rotation, it was found to be six degrees. These minor amounts of molar rotation, together with the insignificant amount of anchorage loss, further demonstrated the ability of this technique to conserve anchorage.

The bone density at the mesial and distal side of the distracted canine did not differ significantly than that in the control side. This might be due to the minimal bone removal during surgery and the fact that bone formed mesial to both canines, at the tension sides, was not affected by the rate of canine retraction in the distraction side. In addition, an increased rate of canine retraction was achieved with a minimal amount of distal surface bone removal from the distracted distal surface.

Root resorption is a frequently reported side effect of orthodontic treatment and has been attributed to a multitude of biologic and mechanical factors [35]. The absence of detectable root resorption in the present study, both visually and densitometrically, could be due to the reduced duration of the treatment. Most studies reported that the severity of root resorption was directly related to treatment duration [36,37,38]. Only a few studies did not support this finding [39,40]. It has been found that 34% of examined teeth showed root resorption after 6–9 months of treatment, whereas at the end of active treatment, lasting 19 months, root resorption increased to 56%. Histologically, 34% and 56% of the examined teeth showed resorbed lacunae after 15 and 20 days of tooth movement, respectively [39,40,41,42].

The panoramic radiographic evaluation after complete canine retraction revealed distal tipping of the canine. The degree of canine tipping was not significantly different between the retracted canine at the experimental side and the retracted canine at the control side. This in contrary to the radiographic findings of Liou and Huang [25] and Sayin et al. [28]. The tipping of the canines at both sides during retraction might be related to the edgewise brackets used in the present study as it had no pre-angulated bracket slot. The retraction of the canine was also accomplished by sliding along a round stainless steel archwire that produced a point of contact, not an area of contact, that enhanced tipping movement of the canine [43,44].

The pocket depth mesial and distal to the distracted canine was not significantly different than that of the canine at the control side. This might be attributed to the simple surgical procedure done in the buccal bone plate compared to the removal of blocks of bone in the other techniques which may affect the periodontal tissue to a great extent.

The direct proportional correlation between the rate of canine retraction, at either the experimental side of the control side, with the other variables including bone density, pocket depth, anchorage loss, and root resorption, was not statistically significant. Nonetheless, it indicated that the increased rate of canine retraction, either with distraction procedures or conventional methods, mostly leads to increased pocket depth, an increased rate of root resorption, and enhanced anchorage loss. Accordingly, the unprecedented increase in the rate of canine retraction by any method should be exercised with caution as it may have a destructive effect on the supporting tissues and the stability of the anchorage unit.

## 4. Materials and Methods

### 4.1. Study Design and Study Subjects

A clinical trial with the split-mouth design was conducted among twenty orthodontic patients recommended for first premolar extraction with an age range from 13 to 21 years, selected from patients seeking orthodontic treatment in private dental clinics, with consent, in Jeddah, Kingdom of Saudi Arabia.

### 4.2. Inclusion Criteria

1. Ethical consent approval
2. Orthodontic treatment entailing the extraction of first premolar teeth.
3. The full eruption of all permanent teeth except third molars.
4. Good oral hygiene.
5. No previous orthodontic treatment.
6. No history of serious medical problems.

### 4.3. Study Procedure

The right side of the maxillary arch on which corticotomy was performed was considered as the experimental group, and the other side without surgical intervention was considered as the control group. All patients were informed about the procedure, and written informed consent was obtained from all the patients or the parents/guardians in case of minors.

For each patient, the following records were taken to satisfy the research design:

1. Extra-oral photographs (frontal (at rest and smile), right and left) before treatment.
2. Intra-oral photographs before treatment (right, left, frontal, upper, and lower).
3. Orthodontic study models before and after canine retraction.
4. Standardized digital lateral cephalometric radiograph before treatment.
5. Digital panoramic radiograph before and after canine retraction.
6. Standardized digital periapical dental radiograph from canines to second premolars on both sides, before treatment, after corticotomy and after completion of space closure.

### 4.4. Orthodontic Appliance

Direct bond slot brackets 0.022”(Victory Series; 3 M unitek, Monrovia, CA, USA) were applied from maxillary 2nd premolar to maxillary 2nd premolar (Canine brackets with hooks) using chemical cure orthodontic adhesive. Banding of 1st molars was done with combination buccal tubes (0.018” × 0.025′’) and 0.045” of the round tube with hooks. Archwire of 0.014-inch NiTi Heat actiavated was used for levelling and alignment. Archwire of 0.018-inch stainless steel was used for maxillary canine retraction. Transpalatal arch appliance fabricated from 0.9mm stainless steel wire soldered to the maxillary first molar bands for reinforcement of anchorage. The maxillary first molar and second premolar were ligated to each other on the right and left side to reinforce the anchorage as well as the four incisors with a stainless-steel ligature wire (0.25 mm) to avoid spacing in the anterior teeth during retraction.

### 4.5. Surgical Procedure

The upper right and left first premolars were extracted according to the treatment plan. After extraction of the upper right first premolar, partial corticotomy was done on the buccal bone plate between the upper canine and the socket of the extracted premolar using small surgical fissure bur under copious saline irrigation. Its length extends to the level of the canine apex (detected by digital dental radiography) with depth and width not more than 1 and 2 mm, respectively. The contralateral side served as control.

### 4.6. Canine Retraction

Canine retraction on both sides was started after 14 days from the surgical procedure using elastic power chain extended between the maxillary canine hook and the maxillary first molar hook. The right and left canines were retracted distally using the same force magnitude of 150 g on average and changed by a new one every two weeks till complete closure of the extraction space in either of the two sides.

### 4.7. Measurements

Measurements of the rate of extraction space closure (canine retraction) was done clinically from the distal end of the canine bracket to the mesial end of the 2nd premolar bracket by using digital dental vernier every two weeks till the closure of the extraction space.

Study model analysis before and after canine retraction by two methods: (a) direct method, done by using digital calliper directly onto the study casts from the cusp tip of the canine to the cusp tip of the second premolar, and (b) cast photocopy.

A digital periapical radiograph was used before and after canine retraction to evaluate the condition of the investing tissue distally and mesially to the retracted canine, pocket depth, and root resorption of the canines.

The bone density was measured using the Digora software. For pocket depth measurements, the longitudinal axis of the canine was marked, then two perpendiculars from the deepest point in the pocket and the cementoenamel junction were drawn to the longitudinal axis, then the distance between the two points on the longitudinal axis was measured

The periapical films of the canines right before the first premolar extractions and at the end of the canine distractions were both projected on a screen and magnified by 10.

The apical root resorption was assessed by the following scores [24]:

- 0 = No apical root resorption
- 1 = Slight blunting of the canine root apex
- 2 = Moderate resorption of the root apex beyond blunting and up to one-fourth of the root length.
- 3 = Excessive resorption of the root apex beyond one-fourth of the root length

The lateral root resorption on the distal side of the canine root was assessed according to the following scores [24]:

- 0 = Smooth lateral root surface and periodontal ligament
- 1 = Slightly irregular lateral root surface; not beyond one-third of the dentine width between the distal side periodontal ligament and pulp chamber
- 2 = Moderate irregular lateral root surface beyond one third and up to two-thirds of the dentine width between the distal side periodontal ligament and pulp chamber
- 3 = Excessive irregularity of the lateral root surface beyond two-thirds of the dentine width between the distal periodontal ligament and pulp chamber.

After the research design was completed, the patients continued their treatment according to their proposed treatment plan.

### 4.8. Statistical Analysis

The data were entered in Microsoft Office, Excel worksheets and analyzed using software IBM SPSS v. 20.0 (IBM Statistics, SPSS, Chicago, USA). The normality of the data was assessed using the Shapiro Wilk test while Levene’s test for equality of error variances was used to analyze the homogeneity of error variances. Descriptive statistics were calculated. Paired-sample *t*-tests were employed to evaluate inter-group differences. Statistical significance was determined at α = 0.05.

## 5. Conclusions

The technique of partial corticotomy of the buccal plate distal to the canine is a straightforward surgical procedure enhancing the rate of canine retraction significantly. Improving the rate of canine retraction in the current study, with the very conservative corticotomy technique, significantly reduce patient complains, anchorage loss, and the positive adverse effects on the teeth investing tissues. The fundamental advantage of the current approach was increasing the rate of canine retraction and, consequently, the overall treatment time.

## Figures and Tables

**Table 1:** Comparison of the rate of canine retraction at the partial corticotomy side (upper right side) and the control side (upper left side).

	Mean	SD	Mean Difference	*T*-Value	*p*-Value
Corticotomy Side	0.70	0.14	−0.11	−2.76	0.01
Control Side	0.59	0.11			

**Table 2:** Comparison of the molar anchorage loss of the upper first molars in the partial corticotomy side and the control side (to the nearest 0.1 mm).

		Mean	SD	Mean diff	*T*-Value	*p*-Value
1st measurement	Corticotomy	0.91	0.49	0.09	0.55	0.58
	Control	1.00	0.54			
2nd measurement	Corticotomy	1.75	0.61	0.25	1.20	0.24
	Control	2.00	0.70			

**Table 3:** Comparison of the premolar anchorage loss of the upper first molars in the partial corticotomy side and the control side (to the nearest 0.1 mm).

		Mean	SD	Mean Diff	*T*-Value	*p*-Value
1st measurement	Corticotomy	0.75	0.68	0.53	1.85	0.072
	Control	1.28	1.09			
2nd measurement	Corticotomy	1.50	0.70	0.50	1.90	0.66
	Control	2.00	0.95			

**Table 4:** Comparison of the amount of canine rotation in the partial corticotomy side and the control side (to the nearest 0.5 degrees).

	Mean	SD	Mean Diff	*T*-Value	*p*-Value
Corticotomy side	15.16	6.67	3.00	1.50	0.14
Control Side	18.16	5.91			

**Table 5:** Comparison of the amount of Molar rotation in the partial corticotomy side and the control side (to the nearest 0.5 degrees).

	Mean	SD	Mean Diff	*T*-Value	*p*-Value
Corticotomy side	6.00	1.41	0.33	0.66	0.52
Control Side	6.33	1.75			

**Table 6:** Descriptive statistics of bone densities in both sides before treatment, after extraction and after the closure of the extraction space (HU units).

		Corticotomy		Control		Mean Difference	*T*-Value	*p*-Value
		Mean	SD	Mean	SD			
Mesial	Before treatment	107.03	15.16	99.86	9.08	−7.17	−1.85	0.078
	After 1st premolar extractions	107.54	12.24	99.78	13.65	−7.76	−1.89	0.066
	After closure of extraction space	95.56	13.88	94.67	8.74	−0.98	−0.27	0.79
Distal	Before treatment	117.18	13.54	110.76	10.43	−6.42	−1.68	0.10
	After 1st premolar extractions	108.86	14.51	99.40	17.64	−9.46	−1.85	0.072
	After closure of extraction space	107.79	13.59	98.33	18.98	−9.46	−1.81	0.079

**Table 7:** Comparison of pocket depth in both sides before treatment and after closure of the extraction space.

		Corticotomy		Control		Mean Difference	*T* Value	*p* Value
		Mean	SD	Mean	SD			
before beginning of canine retraction	Mesial pocket depth	0.66	0.51	0.83	0.75	0.17	0.84	0.41
	Distal pocket depth	1.50	1.22	1.33	0.81	−0.17	0.52	0.61
after the closure of the extraction space	Mesial pocket depth	1.50	0.80	1.83	0.40	0.33	1.65	0.11
	Distal pocket depth	2.66	1.03	2.33	0.81	−0.33	−1.13	0.27
